# Objectively Measured Maternal Supine Sleep and Fetal Growth: A Prospective Cohort Study

**DOI:** 10.1111/1471-0528.70246

**Published:** 2026-04-13

**Authors:** Danielle L. Wilson, Dwayne L. Mann, Irene Szollosi, Leonie Callaway, Alka Kothari, Philip I. Terrill

**Affiliations:** ^1^ Sleep Disorders Centre The Prince Charles Hospital Brisbane Queensland Australia; ^2^ School of Electrical Engineering and Computer Science The University of Queensland Brisbane Queensland Australia; ^3^ Faculty of Health, Medicine and Behavioural Sciences The University of Queensland Brisbane Queensland Australia; ^4^ Women's and Newborn Services Royal Brisbane and Women's Hospital Brisbane Queensland Australia; ^5^ Faculty of Medicine The University of Queensland Brisbane Queensland Australia; ^6^ Department of Obstetrics and Gynaecology Redcliffe Hospital Brisbane Queensland Australia

**Keywords:** back, night‐to‐night variability, phenotype, posture, pregnant, sleep onset, subjective

## Abstract

**Objective:**

To explore the relationship between different definitions of objectively measured supine sleep position during late pregnancy and customised birthweight centile, as a marker of fetal wellbeing.

**Design:**

Prospective cohort study.

**Setting:**

Australian public hospital antenatal clinic.

**Population:**

Eighty‐four pregnant women from 32–36 weeks of gestation.

**Methods:**

Body position was measured in degrees of roll using a tri‐axial accelerometer worn around the abdomen for seven sequential nights. Supine was defined as ‘broad supine’ (0° = fully supine to 45° left/right tilt), and ‘supine low‐tilt’ (0°–15°). Two additional Risk Indices capturing risk proportional to duration spent in each degree of supine‐to‐lateral tilt were developed. Birthweights were converted into customised centiles using GROW software.

**Main Outcome Measures:**

The primary outcome was customised birthweight centile. Secondary outcomes included birthweight, gestational age at delivery, and fetal growth trajectory.

**Results:**

Across 556 nights of data, the median nightly duration of broad supine positioning was 65.9 [25.0, 125.7] minutes compared to only 9.1 [0.7, 24.4] minutes of supine low‐tilt. There was no relationship between customised birthweight centile and time spent in broad supine (*R* ^2^ = 0.0027, *F*(1,82) = 0.22, *p* = 0.64) or supine low‐tilt (*R* ^2^ = 0.0029, *F*(1,82) = 0.24, *p* = 0.63); nor with any supine risk index and customised birthweight centile, birthweight, gestational age at delivery, or fetal growth trajectory.

**Conclusion:**

In this cohort, there was no evidence of an association between objectively measured supine sleep and customised birthweight centile. Yet to be identified mechanisms unrelated to fetal growth may be responsible for supine going‐to‐sleep position being a risk factor for late stillbirth. Further prospective investigation is needed to untangle cause and effect; however, given the low rate of stillbirth, such a study may not be feasible.

## 1 Introduction

Going to sleep on the back during late pregnancy has recently been identified as a modifiable contributor to increased risk of stillbirth after 28 weeks of gestation [1] and low birthweight infants [2, 3]. Subsequently, a number of countries have introduced educational campaigns within maternity healthcare centres to encourage side‐sleeping from 28 weeks of gestation [4, 5, 6, 7].

The purported mechanism behind supine sleep increasing risk of stillbirth from 28 weeks of gestation is that when lying supine, the enlarged uterus may cause compression of the inferior vena cava (IVC) and aorta, resulting in a fall in cardiac output and reduced blood flow to the placenta and fetus [8, 9]. Lying fully supine during wake reduces blood pressure, cardiac output, and uteroplacental blood flow [9, 10, 11], and these effects may persist with a lateral tilt up to 15° [12, 13, 14]. In contrast, a lateral tilt of ≥ 30° may alleviate compression of the inferior vena cava [15], with subsequent elevations in cardiac output [16] and correction of hypotension in some women [17].

While studies linking maternal sleep position to fetal growth restriction and stillbirth are based on the best correlational evidence available, key limitations remain. Firstly, studies have assessed sleep position at the start and end of the night by retrospective self‐report [2, 18, 19, 20, 21, 22], and perception of supine sleep presence/duration can be inaccurate [23, 24, 25]. Second, while a limited number of studies have objectively measured sleep position [26, 27, 28, 29, 30], traditional position sensors used in sleep studies only measure five body positions (left, right, supine, prone and upright), with supine capturing lying fully supine through to 45° left or right lateral tilt. As such, a more precise sensor is required to differentiate between positions where maternal and fetal haemodynamics are known to be different [15, 16]. Finally, our recent multi‐night analysis found that women in the third trimester averaged an hour per night of sleep on their back. However, night‐to‐night variability was substantial [30], with a single night recording unlikely to give a true representation.

As such, this study aimed to investigate the relationship between supine sleep position during pregnancy and fetal growth outcomes, given that fetal growth restriction is one of the strongest risk factors for stillbirth [31]. Using objective measurement of sleeping position as degree of roll from supine, we firstly aimed to develop risk indices incorporating degree of ‘supineness’ and exposure (time) across a week of monitoring. We hypothesised that increased duration of supine body position overnight was related to lower customised birthweight centile, and this relationship would be stronger for risk indices more heavily weighting positions with lower degrees of tilt from fully supine.

## 2 Method

### 2.1 Study Design and Participants

This study was a single centre, observational prospective cohort study conducted between May 2023 and November 2024. Participants were recruited from the antenatal clinic at Redcliffe Hospital, Queensland, and were aged 18 years or older with a singleton pregnancy without known fetal abnormalities or pregnancy complications (including pre‐eclampsia and fetal growth restriction) at the time of recruitment and were non‐smokers. The Human Research Ethics Committee for Metro North Health and Hospital Services approved the study and written informed consent was obtained from all participants. There was no patient or public involvement in the development of this study.

### 2.2 Procedure

High temporal and spatial resolution sleep position was measured for seven consecutive nights at home using actigraphy (GENEActiv, Activinsights Ltd., UK) between 32 and 36 weeks of gestation. After providing training in the use of the device, the GENEActiv was either supplied to the participant at an antenatal appointment or via post.

**FIGURE 1:**
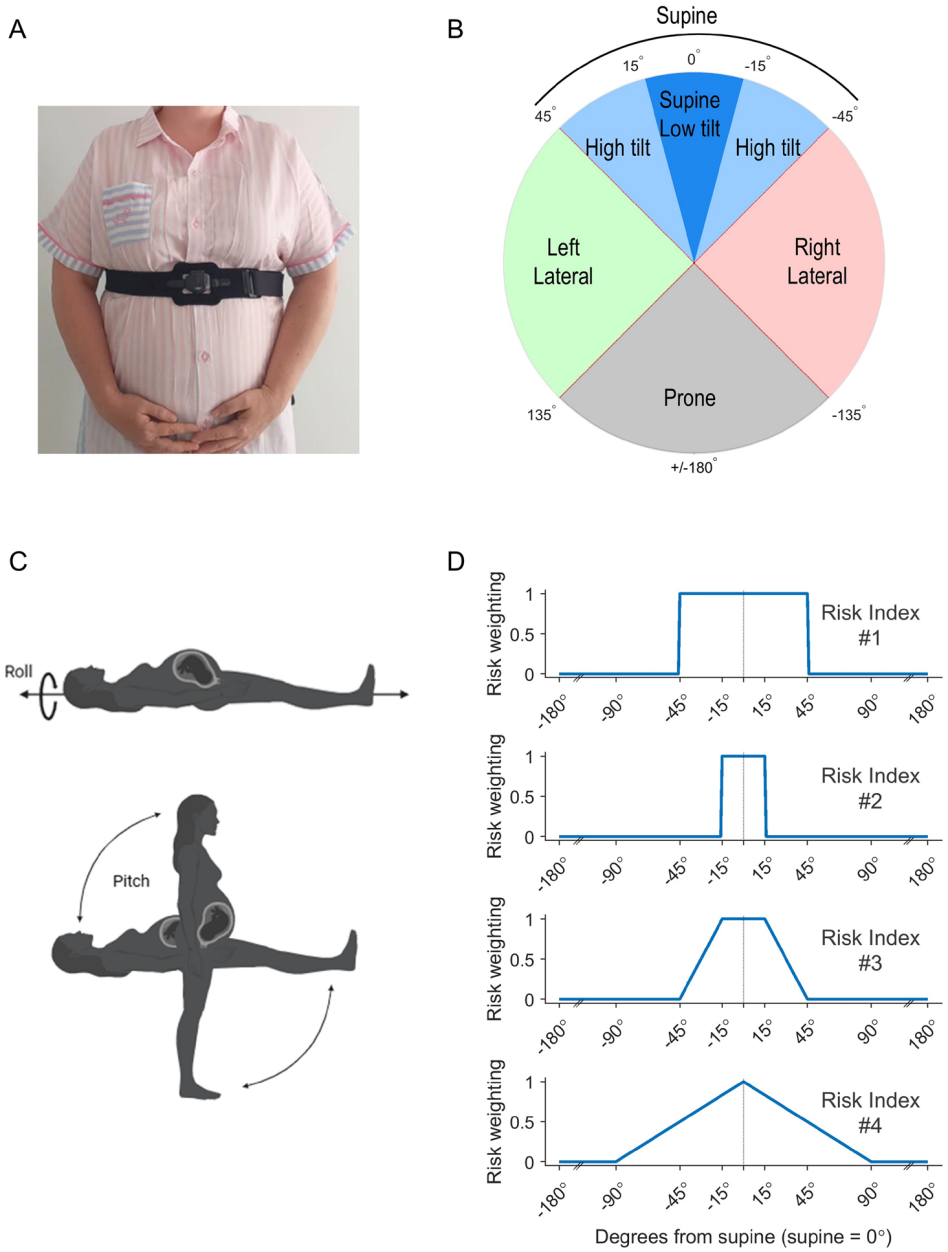
(A) GENEActiv position sensor worn on a belt above the pregnant abdomen, centrally positioned; (B) Continuous roll position categorised into traditional position quadrants (i.e., supine, −45° to 45°; left lateral, 45° to 135°; right lateral, −45° to −135°; and prone 135° to 180° & −135° to −180°) and supine stratified into tilted supine categories (i.e., supine low‐tilt, −15° to 15° and supine high‐tilt, 15° to 45° & −15° to −45°); (C) Orientation and stratification of body roll positions—‘Roll’ was set as rotation about the body's longitudinal axes (with positive values assigned to rotation towards the left position), while ‘Pitch’ was set to capture lying vs upright position; (D) Diagrammatic representation of exposure risk indices. In the subplots for Risk Index #1 and #2, only values within the degree range of interest (0°–45° and 0°–15°, respectively) are counted and are included at their face value. Subplots for Risk Index #3 and #4 show the two novel weightings used, where time at the corresponding degrees from supine is weighted by the indicated risk weighting value.

The GENEActiv is typically worn on the wrist; however, it can be fitted to various body locations. Since advice regarding sleeping position in pregnancy is focused on the potential impact of the weight of the fetus on cardiovascular and hemodynamic function, participants were instructed to self‐apply the GENEActiv using an elasticised belt (designed for the Night Shift Sleep Positioner; Advanced Brain Monitoring Inc.) centrally located around the abdomen positioned above the top of the fundus (Figure 1A). The GENEActiv was applied just before going to bed each night and removed upon waking in the morning. The research team did not provide any sleeping position instructions; however, the antenatal clinic from which participants were recruited provided routine clinical ‘Sleep on Side’ recommendations.

Each morning, participants completed a sleep diary which asked about bedtime, lights off and wake time, perceived sleep duration and quality, along with self‐reported sleep onset position and wake position, and whether they spent time overnight sleeping on their back: (i) none, (ii) yes—less than an hour or (iii) yes—more than an hour (See Supporting Information Page 2–3).

Participants were also asked about additional pillow usage overnight, and to confirm whether the GENEActiv device stayed in the correct location overnight. To examine possible movement of the belt overnight, a subset of 12 participants concurrently wore one GENEActiv on the belt and another adhered with tape for one night.

Demographic and obstetric data were collected at recruitment, including maternal age, parity, gestation and pre‐pregnancy body mass index (BMI). Birth outcomes included maternal complications such as diagnosis of a hypertensive disorder and gestational diabetes, birthweight and gestational age at delivery, collected from hospital records. Data from clinical growth scans were collected where available. The birthweight following delivery and ultrasound estimates of fetal weight were customised for gestational age, maternal height and pre‐pregnancy weight, ethnicity, parity, and fetal sex using the Australian dataset of the GROW software (Bulk Centile Calculator V8.0.6.2; www.gestation.net) [32].

### 2.3 Actigraphy Processing

The detailed methodology for actigraphy processing is available in the Supporting Information Page 4 and Figure S1. In short, accelerometry data was visualised together with sleep diary data to identify each overnight sleep period. Body position was calculated using Euler angles from the raw unfiltered tri‐axial accelerometry data [33, 34] to determine roll and pitch angles in degrees at the device sampling rate (10 Hz) (Figure 1C). The application of the device as a belt has not previously been validated for recognition of position angle, however the device has been extensively tested for activity and movement monitoring while placed at the level of the waist, and our own bench testing determined accuracy of angular data (see Supporting Information Page 6 and Figure S2). As we could not definitively identify when the participant was asleep, we used monitoring time to report the recording period each night. Artefact from substantial movements and/or upright body position (indicating the participant was likely out of bed or not attempting sleep) was removed. Nights when the participant reported the GENEActiv had moved substantially (e.g., > 5 cm from centre) or where monitoring time was < 4 h after artefact removal were excluded from analysis.

Figure 1B demonstrates how roll data was stratified into traditional polysomnography‐based body position categories with 0° = fully supine (‘broad’ supine, −45° to 45°; left lateral, 45° to 135°; right lateral, −45° to −135°; and prone 135° to 180° & −135° to −180°); and novel tilted supine categories (supine low‐tilt, −15° to 15° and supine high‐tilt, 15° to 45° and −15° to −45°). For brevity, broad supine is henceforth referred to as 0°–45°, supine low‐tilt referred to as 0°–15°, and supine high‐tilt referred to as 15°–45°.

### 2.4 Supine Sleep Risk Indices

We developed four indices to quantify risk associated with supine sleep (Figure 1D). Informed by previous literature regarding the impact of lateral tilt on maternal and fetal haemodynamics, Index #1 and Index #2 quantify the unweighted time spent in broad supine (0°–45°) and supine low‐tilt (0°–15°) respectively. We also calculated two measures weighted by body roll angle. Risk Index #3 builds upon studies reporting minimal change in relevant haemodynamics with body roll between 0° to ±15° [12, 13, 14], with haemodynamic improvement as tilt increases from 15°–45° [15] (decreasing weight until = 0 at 45°) [12, 13, 15]. Risk Index #4 is inspired by the haemodynamic contrast between supine (0°) and lateral (90°) positions [9, 35], and describes relative exposure as a decreasing linear relationship (weight = 1 at 0°, weight = 0 at 90°). The duration (minutes) and percentage of total monitoring time (TMT) spent in each supine category, and each Risk Index, were calculated per night and averaged across the week. Additional details on development of (these and other) risk indices are provided in the Supporting Information (Page 7 and Figure S3).

### 2.5 Outcomes

The primary outcome was customised birthweight centile calculated using the Australian dataset of the GROW software [32]. Secondary outcomes included birthweight, gestational age at delivery and fetal growth trajectory.

### 2.6 Sample Size

We have previously shown that the median percentage of supine sleep across a week in late pregnancy was related to lower birthweight centile (*r*_*s*_ = −0.45, *p* = 0.006), which was attenuated after adjustment for confounders [30]. To detect an effect size of *r* = 0.45, a sample size of 36 is required for a power of 80% and an alpha level of 0.05. To be conservative and account for potential attenuation of the strength of this relationship with adjustment for confounders [30], we aimed to detect at least a medium effect size (*r* = 0.30), which increased the required sample size to 85.

### 2.7 Statistical Analysis

Statistical analyses were performed with R Statistical Software (v4.3.1) [36]. The data that support the findings of this study are openly available at Figshare [37]. Data are presented as means ± standard deviation or median (interquartile range) for non‐normally distributed variables. A two‐sided *p*‐value of < 0.05 was considered to indicate statistical significance.

Indices of body position data were analysed across all nights and as a nightly average (sum of data from all valid nights divided by number of valid nights monitored) per participant and presented as descriptive statistics. Time spent in a left versus right supine tilt was compared using a Wilcoxon signed‐rank test. The roll position at participant‐declared sleep onset was determined using the majority (mode) position from 5 min at each onset timepoint.

For secondary analysis of fetal growth trajectory, only growth scans after 24 weeks of gestation and at least six weeks prior to delivery were included. For participants with more than one scan during this time, the scan most immediately prior to the week of sleep position monitoring was used. To facilitate standardised comparison, a rate of change in customised centile per week was calculated.

Linear regression was used to assess relationships between supine risk indices and customised birthweight centile, birthweight, gestational age at delivery and change in customised centile per week from growth scan to delivery, with adjustment for risk factors related to stillbirth and fetal growth (maternal age, BMI, parity, GDM and hypertensive disorders of pregnancy) [31]. Logistic regression was used to calculate the odds of small‐for‐gestational age (SGA), defined as < 10th customised birthweight centile, based on metrics of supine sleep. Due to positive skewness, all supine measures were Box Cox transformed and standardised to *z*‐scores with a mean value of 0 and standard deviation of 1. Independent sample *t*‐tests were used to compare customised birthweight centile based on self‐reported supine sleep.

## 3 Results

### 3.1 Participants

Eighty‐eight women were recruited to the study and 85 completed data collection. One participant delivered at 44^+2^ weeks of gestation and was excluded from analysis (customised birthweight centile could not be calculated). Twenty‐eight nights (4.8%) were excluded due to device movement or insufficient monitoring time (*N* = 26 and 2 respectively). Of the 84 participants included in the analysis, 60 completed seven nights of monitoring, 17 completed six nights, 6 completed five nights, and one person completed four nights, for a total of 556 nights of data. Participant characteristics are presented in Table 1.

**TABLE 1:** Participant characteristics.

	Average	Range
Age (years)	30.4 ± 4.3	21–41
BMI (pre‐pregnancy) kg/m^2^	25.7 (22.3, 30.5)	18.7–51.9
Gestational age@position monitoring (weeks^days^)	33^+2^ (32^+3^, 34^+4^)	32^+0^–36^+2^
Nulliparous	49 (58.3%)	
GDM@position monitoring & delivery	13 (15.5%)	
HDP@position monitoring	2 (2.4%)	
HDP@delivery	7 (8.3%)	
Ethnicity		
European	78 (92.9%)	
Filipino	2 (2.4%)	
Indian	1 (1.2%)	
Papua New Guinean	1 (1.2%)	
Māori	1 (1.2%)	
Torres Strait Islander	1 (1.2%)	

*Note: N* = 84 participants. Values presented as M ± D, Median (IQR) or *n* (%).

Abbreviations: BMI, body mass index; GDM, gestational diabetes mellitus; HDP, hypertensive disorder of pregnancy.

### 3.2 Precise Overnight Body Position

**FIGURE 2:**
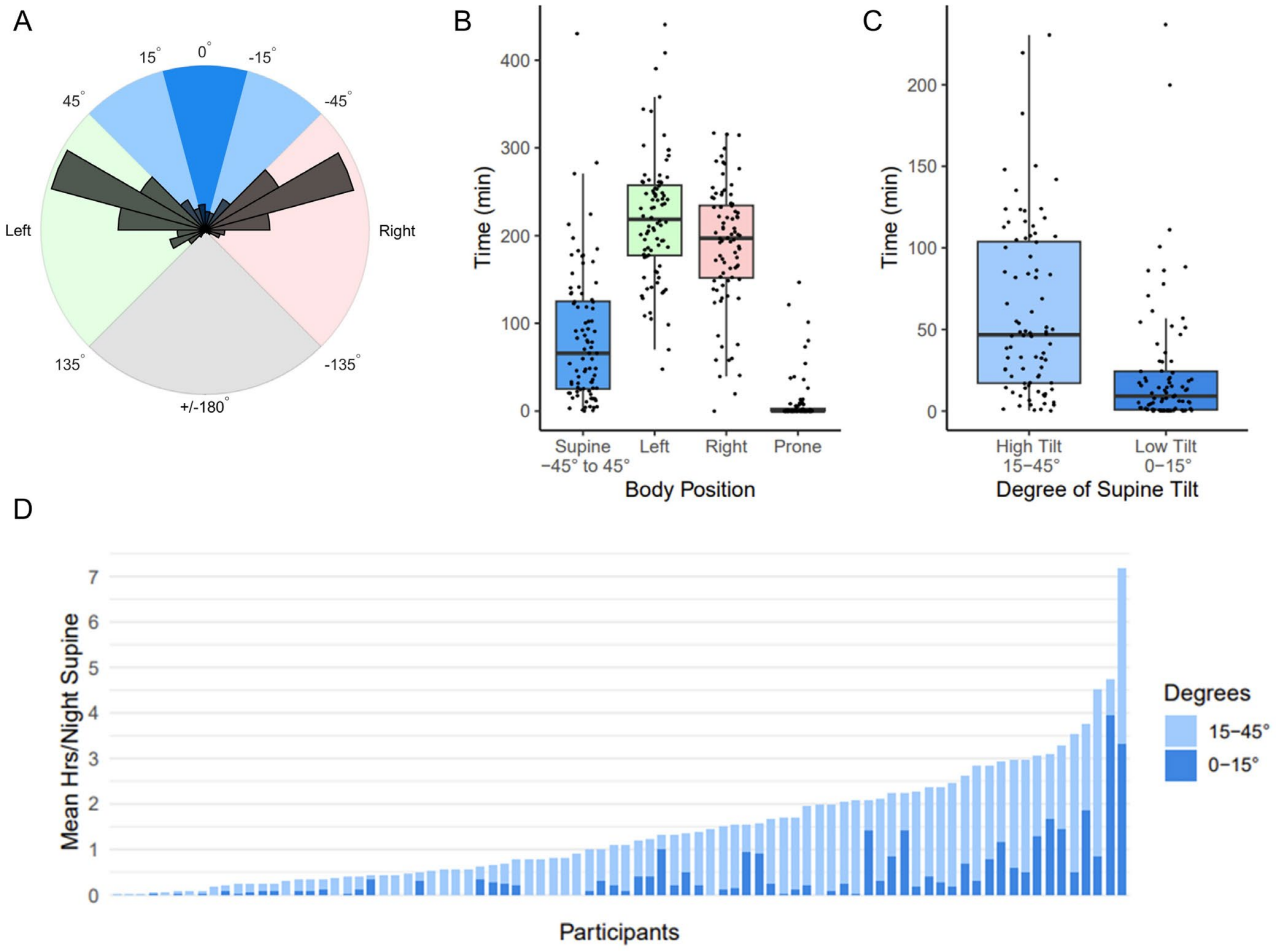
(A) Circular histogram of all 556 nights of data aggregated. Each wedge indicates the degree of tilt from supine = 0°, with the radial length of the wedge representing the proportion of time spent in this body position across the week; (B) Average minutes per night across the week of monitoring spent in each body position for all participants, with (C) supine body position divided into the high‐tilt and low‐tilt definitions; (D) Mean number of hours per night spent in high‐tilt (15°–45°) vs low‐tilt (0°–15°) supine body position across all participants, ordered from least to most total supine monitoring time. Seventeen percent of supine time was spent in the low‐tilt position.

Figure 2 details the duration spent in each degree of roll (Figure 2A) and discrete body position (Figure 2B,C) per night across the week. The average monitoring time per night was 8.4 ± 0.8 h. On average, pregnant women spent 65.9 (25.0, 125.7) min per night (13.6% TMT (4.9, 25.3)) lying on their back by the broad definition of 0°–45° (Figure 2B) but spent just 9.1 (0.7, 24.4) min per night in the low‐tilt definition of 0°–15° (1.9% TMT (0.1, 4.8) and 17.3% (5.2, 38.1) of broad supine; Figure 2C,D). The most frequent overnight position was left lateral (43.2% TMT (34.1, 50.9)), which was marginally more than right lateral (40.2% TMT (29.9, 46.0)). Within the broad supine definition, there was no difference between proportion of time spent in a left or right tilt (%TMT; left = 6.7% (1.2, 12.7) vs. right = 5.6% (2.1, 12.0), *p* = 0.70).

There was substantial inter‐ and intra‐participant variability in nightly supine position time (See Supporting Information Page 9 and Figures S4 and S5). The median within‐person difference between minimum and maximum supine time was 97.5 min (61.9, 143.0) for broad supine (0°–45*°*) and 37.5 min (3.8, 71.3) for low‐tilt supine (0°–15°).

Participants reported using additional back support pillows on 256 nights, with additional leg or abdominal support pillows on 217 nights. Time spent in the supine position on these nights did not differ from nights with no additional pillows (*n* = 63 nights; Supporting Information Page 12 and Figure S6).

Data from 11 participants concurrently wearing GENEActiv on the belt and another adhered with tape for one night indicated minimal movement of the belt overnight (group mean for Spearman's rho = 0.93; Supporting Information Page 13 and Table S1).

### 3.3 Supine Body Position Overnight and Fetal Growth Outcomes

The average gestational age at delivery was 39.6 ± 1.4 weeks, with an average birthweight of 3489.0 ± 449.6 g and customised birthweight centile of 48.5% ± 29.1%. Nine (10.7%) infants were classified as small for gestational age (< 10th customised centile), with another eight (9.5%) classified as large for gestational age (> 90th customised centile).

**FIGURE 3:**
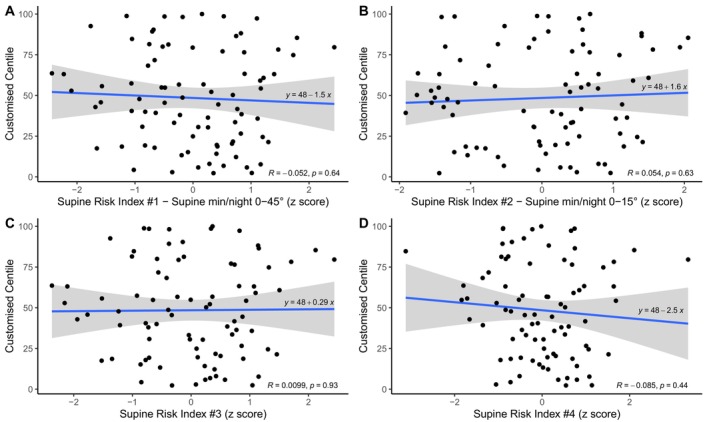
Average time (mins) spent in the supine position overnight versus customised birthweight centile for (A) Risk Index #1 Broad Supine = 0°–45°, (B) Risk Index #2 Supine Low‐Tilt = 0°–15°; and (C) Risk Index #3 and (D) Risk Index #4 versus customised birthweight centile. Supine (mins) and Risk Indices were Box Cox transformed and standardised to *z*‐scores.

There were no relationships between the average duration per night (mins) across the week spent in the supine position and customised birthweight centile, when defined as either broad supine (0°–45°, Risk Index #1, *R* ^2^ = 0.003, *F*(1,82) = 0.22, *p* = 0.64) or supine low‐tilt (0°–15°, Risk Index #2, *R* ^2^ = 0.003%, *F*(1,82) = 0.24, *p* = 0.63; Figure 3 and Table 2). There were also no relationships between supine body position overnight and birthweight or gestational age at delivery (Table 2), with all standardised beta values (*β*) of 0.13 or less. Adjustment for the stillbirth risk factors of age, pre‐pregnancy BMI, parity, GDM and hypertensive disorders did not change these relationships (Table 2, full model Tables S2 and S3). Similarly, Risk Index #3 and Risk Index #4 weighted by duration spent in different degrees of supine‐to‐lateral tilt were unrelated to customised birthweight centile (*β* = 0.01 and −0.08), birthweight (*β* = 0.06 and −0.07) or gestational age at delivery (*β* = 0.17 and 0.07; Figure 3, Table 2, Tables S4 and S5, additional exploratory Risk Indices in Tables S6–S8).

**TABLE 2:** Regression coefficients for predicting customised birthweight centile, birthweight and gestational age at delivery from indices of overnight supine body position.

	Unadjusted		*p*	Adjusted		*p*
	Coeff (95% CI)	Beta		Coeff (95% CI)	Beta	
Customised birthweight centile (%)						
0°–45° supine min/night (Risk Index #1)	−1.51 (−7.88, 4.87)	−0.05	0.64	−1.50 (−7.74, 4.74)	−0.05	0.63
0°–15° supine min/night (Risk Index #2)	1.56 (−4.82, 7.94)	0.05	0.63	−0.01 (−6.40, 6.37)	0.00	0.99
Risk index #3	0.29 (−6.10, 6.67)	0.01	0.93	−0.21 (−6.48, 6.07)	−0.01	0.95
Risk Index #4	−2.47 (−8.83, 3.90)	−0.08	0.44	−2.12 (−8.37, 4.13)	−0.07	0.50
Birthweight (g)						
0°–45° supine min/night (Risk Index #1)	−11.36 (−110.09, 87.37)	−0.03	0.82	2.81 (−93.13, 98.76)	0.01	0.95
0°–15° supine min/night (Risk Index #2)	36.63 (−61.80, 135.07)	0.08	0.46	23.53 (−72.67, 119.72)	0.05	0.63
Risk index #3	26.14 (−72.45, 124.74)	0.06	0.60	30.18 (−65.46, 125.83)	0.07	0.53
Risk Index #4	−30.85 (−129.38, 67.67)	−0.07	0.54	−7.43 (−102.65, 87.79)	−0.02	0.88
Gestational age at delivery (days)						
0°–45° supine min/night (Risk Index #1)	1.29 (−0.82, 3.41)	0.13	0.23	1.25 (−0.84, 3.33)	0.13	0.24
0°–15° supine min/night (Risk Index #2)	1.15 (−0.97, 3.27)	0.12	0.28	1.54 (−0.55, 3.62)	0.16	0.15
Risk index #3	1.60 (−0.50, 3.71)	0.17	0.13	1.64 (−0.43, 3.71)	0.17	0.12
Risk Index #4	0.69 (−1.44, 2.81)	0.07	0.52	0.80 (−1.28, 2.88)	0.08	0.45

*Note:* Adjusted for age, nulliparity, gestational diabetes mellitus, hypertensive disorders of pregnancy, and additionally adjusted for body mass index for birthweight and gestational age at delivery. Supine variables Box Cox transformed and standardised to *z*‐scores—coefficient represents change in outcome given one SD increase in supine variable.

Given the argument that customisation of birthweight centiles may mask or normalise pathologies relating to ethnicity or maternal BMI for example, supplementary analysis was performed using the non‐customised INTERGROWTH 21st birthweight centiles [38] with similar results (Table S9).

There was no increased odds of delivering an SGA infant for a standard deviation increase in the average time spent in the supine position overnight (Risk Index #1: OR = 1.48 (0.73, 3.28), *p* = 0.30; Risk Index #2: OR = 1.25 (0.62, 2.68), *p* = 0.53; Risk Index #3: OR = 1.42 (0.70, 3.05), *p* = 0.35; Risk Index #4: OR = 1.32 (0.66, 2.67), *p* = 0.43).

Within the 39 participants with growth scans available, there was no relationship between either Risk Index #1 (time spent 0°–45° supine) or Risk Index #2 (time spent 0°–15°) and change in customised birthweight centile per week between the growth scan and birth (*R* ^2^ = 0.054, *F*(1,37) = 2.13, *p* = 0.15; *R* ^2^ = 0.025, *F*(1,37) = 0.94, *p* = 0.34 respectively, Figure S7). Using a median cut‐off of 66 min per night in the broad supine position, there was no difference in growth trajectory between the participants with above‐ versus below‐median supine duration (change in customised birthweight centile per week: −1.4 (3.0) versus −3.3 (3.2), *d* = 0.61, *p* = 0.07; larger fall in growth trajectory in the below‐median supine group).

### 3.4 Self‐Reported Supine Position and Birthweight

Due to only four nights of self‐reported supine sleep onset (0.7%), we could not investigate the relationship between self‐report supine sleep onset and infant birthweight outcomes. Twenty‐two participants had objectively measured supine positioning at their reported sleep onset time at least once in the monitoring week (across 32 nights). There was no difference in customised birthweight centile between these participants and those who always started the sleep period laterally (50.4 ± 32.0 vs. 47.8 ± 28.2, *d* = 0.09, *p* = 0.74) or between participants who self‐reported any sleep on their back overnight for at least half of nights monitored (43.0 ± 30.1, *n* = 33) versus those who do not usually report sleeping on the back (52.0 ± 28.2, *n* = 51, *d* = 0.31, *p* = 0.18).

## 4 Discussion

### 4.1 Main Findings

This study aimed to explore the impact of supine sleep during pregnancy on customised birthweight centile. We found that supine positioning overnight, by its traditional broad definition (0°–45°), occurs during pregnancy with half of all recorded nights containing at least 1 h of supine time. However, high‐precision measurement revealed that substantially less time (9.6 min on average) is spent in low‐tilt supine positions (0°–15°), in which the hemodynamic consequences of the gravid uterus on uteroplacental blood flow would be expected to be worse. Despite this, our hypothesis that supine sleep would be related to lower customised birthweight centiles was not supported regardless of supine definition or risk index used. Similarly, we found no relationship between supine positioning and birthweight, gestational age at delivery, or fetal growth trajectory across late pregnancy.

### 4.2 Implications

Policy and maternal education programs encouraging avoidance of supine sleep during late pregnancy have been widely adopted [4, 5, 6, 7, 39, 40, 41], and were formulated on the best available evidence at that time. In particular, a meta‐analysis of case–controlled studies showed supine going‐to‐sleep position had an odds ratio of late‐term stillbirth of 2.63 relative to left side [1], and other studies have shown associations between supine sleep and low birthweight infants [2, 3], and fetal brain sparing [42]. We also previously showed that sleep position in late pregnancy was related to reduced birthweight when objectively measured on a single night [26] and across a week (in a smaller sample) [30]. In contrast, other studies using a single night of sleep position monitoring have found no relationship between supine sleep and birthweight centile [43], fetal growth velocity [27], or a composite measure of stillbirth, small for gestational age and gestational hypertensive disorders [28]. We aimed to strengthen the current predominantly self‐report based evidence by understanding the underlying mechanisms and objectively measuring and characterising sleep position behaviour across multiple nights. However, our data did not support a relationship between supine sleep and customised birthweight centile, birthweight or fetal growth trajectory, with low to very low effect sizes demonstrated. Our novel data demonstrated large nightly variability across and within individuals, for both degrees of roll and duration; and we developed indices ascribing a ‘supine risk’ to each participant based on purported pathophysiological mechanisms in literature to determine whether certain body positions relate to higher risk of adverse fetal outcomes. However, irrespective of how we quantified risk, there was no signal of a clinically important relationship with measured fetal outcomes.

There are several possible explanations for the differences between our findings and previous studies. Firstly, in contrast to the meta‐analysis of case–control studies [1], we used customised birthweight centile rather than stillbirth as the primary outcome because the low frequency of stillbirth would require an unfeasibly large sample size in the required prospective study design [39]. However, fetal growth restriction is one of the strongest risk factors for stillbirth [31], and it is expected that a pathophysiologically significant effect would manifest in changes to birthweight. Second, since going to sleep on the back was only reported four out of 556 nights (despite being objectively measured on 32 nights across 22 participants), we could not replicate previous work by Anderson et al. [2] showing an association between self‐reported supine sleep and customised birthweight centile. Third, supine sleep is not well‐defined in the supporting literature [1]. The physiological mechanisms impacting maternal and fetal haemodynamics during wake differ depending on the degree of supine‐tilt [9, 10, 11, 14, 15, 35]. To date, there is no direct physiological evidence for whether a higher‐tilted position during sleep offers protection from the negative effects associated with supine positioning during wake. Lastly, our objective evaluation may add to speculation that the previously reported associations between sleep position and fetal wellbeing are influenced by other factors rather than true physiology [44].

Women recruited as participants in this study received routine clinical education encouraging side sleeping. The meta‐analysis evidence behind these recommendations reported supine going‐to‐sleep in 4.5% of their sample (comprised of 67 (7.9%) stillbirth cases and 73 (3.2%) liveborn controls) [1]. In contrast, this and our previous study [30] showed a discrepancy between self‐reported supine going‐to‐sleep on only 0.6%–0.8% of nights versus objectively measured supine sleep onset on 6.8%–7.9% of nights. Based on this, supine sleep onset was likely underestimated in data used to support policy [2, 18, 19, 20, 21, 22, 39, 40], and the supine exposure in our study was low. Indeed, very little time was spent tilted within 15° of absolute supine (0°), and perhaps supine exposure was not high enough to lead to changes in fetal outcomes. Even so, participants with the longest duration of completely supine sleep had well‐grown babies.

The current study is the largest investigation of sleep position in pregnancy and fetal growth using precise multi‐night recordings to objectively capture sleeping position patterns and was sufficiently powered to find at least a moderate (*r* = 0.30) effect size. While we were underpowered to support small effect sizes, our data suggest that indices of supine positioning overnight may only explain 0.3% of the variance in birthweight centile. If, as previous data suggests [1], supine sleep is associated with increased risk of stillbirth, we speculate that it is likely through another pathway unrelated to or interacting with fetal growth or another fetal vulnerability. For example, the collateral venous system, and in particular, the azygos system provides an alternative route for blood flow back to the systemic circulation in the event of IVC obstruction [9, 45, 46]. It has been posited that anatomical variations of the abdominal portions of the azygos system that do not support this compensatory pathway could be a risk factor for stillbirth in an already compromised fetus [46]. Similarly, supine sleep may be an interactive risk factor of stillbirth where this anatomical variation is present.

### 4.3 Strengths and Limitations

The key strengths of our study were objective multi‐night recording of sleep position in degrees of roll in the participant's home, thereby representing typical behaviour throughout the third trimester. Indeed, 86% of participants reported that the week of monitoring was typical of their sleep behaviour during the third trimester. In this cohort we demonstrated that most pregnant women have variable amounts of time spent in supine sleep across the week, with an average difference of 97.5 min between the lowest and highest duration of 0°–45° supine. Consequently, an isolated night of measurement is unlikely to capture longer‐term sleep behaviours that may drive important pregnancy outcomes [28, 43].

Given much of the existing data is based on self‐report of sleep onset position, we believed it was valuable to look at how well people could recall body position overnight. This may also be important as the initial sleep onset position may not always be the dominant position overnight [26, 27]. Our previous work showed that women in late pregnancy spend an average of 21.2% of the night awake, and change body position an average of 9.7 times per night [26]. A change in body position generally aligns with a wake period overnight, and therefore assessing whether someone can remember their overnight sleep position may be useful for future studies investigating the relationship between sleep position and fetal wellbeing across larger samples.

However, our study also had some limitations. Firstly, our efforts to obtain highly detailed longitudinal sleep position data meant a trade off in sample size given the resources available for the study, and hence our study was not powered adequately for small effect sizes. Secondly, we did not objectively measure sleep alongside the body position recordings. Given participants performed recordings in their own homes across a week, polysomnography was not feasible for these heavily pregnant women. Secondly, there are no commercial sleep position measurement devices designed for use in pregnancy. We positioned the sensor at the top of the fundus, with a focus on the potential impact of the gravid uterus on cardiovascular and haemodynamic function when lying supine. Extra quality control (see Supporting Information) gave us confidence on the validity of our measure.

By excluding those with known fetal growth restriction, it is possible we introduced bias into the study. However, it is likely that the physiological mechanisms causing vascular flow changes with supine positioning would be more prominent later in pregnancy, rather than before recruitment to the study. Given the sample size, smoking was also excluded to reduce possible confounding reasons for low birthweight other than sleep position. Nevertheless, the exclusion of smokers and those with pregnancy complications at the time of recruitment, and the high proportion of participants with European ethnicity, may limit the generalisability of our findings to low‐risk pregnancies only.

Finally, birthweight and birthweight centile represent only the final size of the infant. Limited resources meant we were unable to conduct growth scans as part of the study protocol, and as such, analysis of fetal growth trajectory was only possible where clinical data was available. As ultrasound predictions of absolute fetal size are subject to error [47, 48], they may be more useful for assessing relative change over time. Many important factors contribute to fetal growth, and a more detailed investigation could identify infants with a fall in fetal growth trajectory independently related to sleeping position. These infants may be at higher risk of perinatal mortality than infants that were consistently SGA with appropriate forward growth trajectory [49].

## 5 Conclusion

Irrespective of the supine definition, we could not support an association between supine position overnight during pregnancy and customised birthweight centile, birthweight, gestational age at delivery, or fetal growth trajectory in this low‐risk cohort. Our findings point to some other mechanism or interaction with fetal growth or vulnerability being responsible for the evidence supporting sleep‐on‐side policy. However, it seems unlikely that future clinical trials to determine if and how this policy has reduced the risk of low birthweight or stillbirth in late pregnancy will be feasible.

## Author Contributions

**Danielle L. Wilson:** conceptualisation, methodology, validation, formal analysis, investigation, writing – original draft, writing – review and editing, visualisation, project administration, funding acquisition. **Dwayne L. Mann:** software, validation, data curation, resources, writing – review and editing, visualisation. **Irene Szollosi:** conceptualisation, methodology, resources, writing – review and editing, supervision, funding acquisition. **Leonie Callaway:** methodology, writing – review and editing, supervision, funding acquisition. **Alka Kothari:** methodology, resources, writing – review and editing, supervision, funding acquisition. **Philip I. Terrill:** conceptualisation, methodology, resources, writing – review and editing, supervision, funding acquisition. All authors have seen and approved this manuscript.

## Funding

This study was funded by a Metro North Health Collaborative Grant CRG‐413‐2022 (D.L.W.). D.L.M. received salary report from National Health and Medical Research Council of Australia Ideas Grant 2001729 and National Health and Medical Research Council of Australia EU‐Horizons Grant 2007001.

## Disclosure

The authors have nothing to report.

## Ethics Statement

The Human Research Ethics Committee for Metro North Health and Hospital Services approved the study and written informed consent was obtained from all participants (HREC2022/MNHA/88879, approved 30 November 2022).

## Conflicts of Interest

The authors declare no conflicts of interest.

## Supporting information


**Figure S1:** Demonstration of gimbal lock experienced by our determination of roll angle.
**Figure S2:** Demonstration showing angular data (specifically Roll) resolved from raw accelerometry. Bench test shows two devices attached to gimbal mount, while moving in Roll orientation 15° steps (from 0° to 120°), and repeated at increasing levels of tilt (0° to 50°, increments of 10°). Top subplot shows raw data from device #1. Middle subplot shows processed Roll data from device #1. Bottom subplot shows processed Roll data from device #2.
**Figure S3:** Diagrammatic representation of exposure risk indices used in additional analyses. A = Risk Index A, B = Risk Index B, C = Risk Index C, D = Risk Index D, E = Risk Index E, F = Risk Index F, G = Risk Index G.
**Figure S4:** Hours per night of supine positioning defined as (A) 0°–45° and (B) 0°–15° with each vertical line and associated dots representing each night for each participant. Participants are plotted in same position on *x*‐axis on each figure, from lowest to highest average time supine 0°–45° per night. Orange dots represent median for each participant.
**Figure S5:** Absolute difference in supine duration overnight between the mean of seven nights monitored within each participant versus the mean of a reduced number of nights, for Broad Supine and Supine Low‐Tilt. The number of nights monitored is chronological for each participant i.e., 1 = the first night monitored, 4 = mean of the first four nights monitored. The absolute difference from mean supine duration over seven nights increases as the number of nights of monitoring decreases, indicating that fewer nights of monitoring provide less precision.
**Figure S6:** Time spent in the supine position defined as (A) 0°–45° and (B) 0°–15°, for nights using a pillow to support the back (*n* = 256), nights using other additional pillows between the knees/under the abdomen (*n* = 217) and nights using no additional pillows (*n* = 63).
**Table S1:** Per participant details on belt versus tape placement of monitoring device.
**Table S2:** Relationship between Supine Risk Index #1 (duration broad supine per night 0°–45°) and fetal growth outcomes.
**Table S3:** Relationship between Supine Risk Index #2 (duration supine low‐tilt per night 0°–15°) and fetal growth outcomes.
**Table S4:** Relationship between Supine Risk Index #3 and fetal growth outcomes.
**Table S5:** Relationship between Supine Risk Index #4 and fetal growth outcomes.
**Table S6:** Regression coefficients for predicting customised birthweight centile from supplementary risk indices of supine position.
**Table S7:** Regression coefficients for predicting birthweight from supplementary risk indices of supine position.
**Table S8:** Regression coefficients for predicting gestational age at delivery from supplementary risk indices of supine position.
**Table S9:** Regression coefficients for predicting 21st INTERGROWTH birthweight centile from key indices of supine position overnight.
**Figure S7:** Change in customised birthweight centile per week between growth scan and birth, with no relationship with average time spent in (A) Risk Index #1 Broad Supine = 0°–45° or (B) Risk Index #2 Supine Low‐Tilt = 0°–15° overnight; and (C) no difference in growth trajectory between the participants with above‐median (≥ 66 min = High Supine) versus below‐median (< 66 min = Low Supine) duration in the supine (0°–45°) position (change in customised birthweight centile per week = −1.4 (3.0) vs. −3.3 (3.2), *p* = 0.07). Supine mins were Box Cox transformed and standardised to *z*‐scores.

## Data Availability

The data that support the findings of this study are openly available in Figshare at figshare.com, available at https://doi.org/10.6084/m9.figshare.31428281.
