# Supplementary material for: Objectively Measured Maternal Supine Sleep and Fetal Growth: A Prospective Cohort Study
Source: BJOG. 2026 Apr 13;133(10):1905–15. doi: 10.1111/1471-0528.70246 (PMC13485437; doi:10.1111/1471-0528.70246)
Supplement: Supplementary file 1 [file BJO-133-1905-s001.docx]

Objectively measured maternal supine sleep and fetal growth: a prospective cohort study.

Wilson DL, Mann DL, Szollosi I, Callaway L, Kothari A, Terrill PI

**Supplement**

# Supplementary Methods

**Sleep Diary - Night 1**	Last night’s date: ______________

1. Did you have any naps yesterday?	Y	/	N

- If so, for how long?

______________________________________________________

2. What time did you get into bed last night? _______________________
3. What time did you turn out the lights and try to sleep? ____________
4. What time did you wake up? __________________
5. How long did it take you to fall asleep? ___________mins
6. How long do you think you slept for? ________hrs _________mins
7. After falling asleep, how many times did you wake overnight? ________
8. Total number of minutes awake overnight? _______________mins
9. How many times did you wake for the following reasons?

- Toilet ___________________
- Child/Bed Partner ___________________
- Discomfort/Physical Complaint ___________________
- External Noises ___________________
- No known reason ___________________
- Other ___________________

10. What would you rate the quality of your sleep,

with 1 = very poor to 10 = very good? _______________________

11. What would you rate your alertness on waking,

with 1 = very poor to 10 = very good? _______________________

12. What position did you go to sleep in?

□ Left	□ Right	□ Back	□ Front

13. What position did you wake up in?

□ Left	□ Right	□ Back	□ Front

14. Did you sleep on your back last night?

□ No	□ Yes - less than 1 hour	□ Yes - more than 1 hour

15. Did you use any additional pillows last night?	Y	/	N

- If so, describe?

______________________________________________________

______________________________________________________

______________________________________________________

16. Did the position sensor stay in the correct position all night? Y	/	N

- If not, what happened and when?

______________________________________________________

______________________________________________________

______________________________________________________

## Actigraphy Processing

Acceleration data was extracted (via proprietary device PC software, V3.3) and subsequently converted to a simple time series (date, time, and acceleration in x, y, z maintained at the original sampling rate of 10Hz) via MATLAB (R2022b, Natick, USA). Additionally, light and temperature data were extracted, and synchronised with the acceleration data. These data (full recording duration) were then visualized together with the associated sleep diary data, providing opportunity to identify and investigate each declared overnight sleep period in greater detail. Within each overnight segment, the diary data (time to bed, lights out, estimated sleep onset, and wake time), x-y-z acceleration data, and the calculated body position (as roll and pitch angles) in degrees were displayed.

Body position (roll) was calculated using Euler angles from the raw unfiltered tri-axial accelerometry data;^1,2^ while this method provides reliable and accurate assessment of position, it is limited by gimbal lock, whereby accuracy across roll or pitch is diminished when one or other axes approach angles +/-90 degrees.

We calculated roll (expressed in degrees) via the four quadrant inverse tangent (i.e., function ‘atan2d’ within MATLAB). This function takes parameters *a* and *b*, where, based on the device orientation while located on our participants, *a* is simply the accelerometer X data, and *b* is given by: -sign(Z) × sqrt( (Y^2^+Z^2^) ); where X, Y and Z represent the corresponding accelerometer data.

Critically, terms *a* and *b* must be real, however, when roll angles approach 90 degrees (either left or right) and together with variable device pitch, the values of Y and Z can approach zero, whereupon the function is not defined, and results in a step in the output (See Figure S1). Importantly, this phenomenon does not affect the roll angles that are of most interest in the current study (i.e., those <|45°|). Finally, despite the identified step in calculated roll angle at ~|90|°, this will not impact our measurements of time (or proportion of time) in lateral positions (left, 45° to 135°; and right, -45° to -135°) as the output is captured within the respective ranges.

Whilst participant sleep diary was used alongside actigraphy, we could not definitively identify sleep periods. Accordingly, we use monitoring time to report the total recording period each night. While monitoring time was initially guided by participant diary responses, it was adjusted using contextual information (such as light, temperature, and overall activity) to capture the overnight rest period. Periods of artefact, such as obvious substantial movement and/or upright body position (indicating the participant was likely out of bed or not attempting sleep) were marked and excluded from subsequent analysis. Nights when the participant had reported that the GENEActiv had moved substantially (e.g. >5cm from centre) were excluded from analysis, as were nights with <4 hours monitoring time after artefact removal.

*Figure S1. Demonstration of gimbal lock experienced by our determination of roll angle.*

## Bench Testing for Actigraphy Device Worn on a Belt

The application of the actigraphy device on a belt has not previously been validated for recognition of position angle. The device has however been extensively tested in free-living patient samples and has obtained clinical-grade validation for activity and movement monitoring while placed on either the wrist, hip/waist or thigh. This validation extends from quantifying gross motor movements, through to detection of fine motor movements such as tremors, but also to observing and characterising sedentary behaviours. Building upon the proven capabilities of the device, we utilised the available unfiltered raw acceleration data to determine angular data (Roll and Tilt), given the known device orientation upon the person, using standard formulae.

To confirm our determination of angular data were accurate, we also conducted a simple bench test. Two devices were attached to a gimbal setup, whereupon we recorded data across a range of actual roll values (from 0° through to 120° in 15° steps) while at increasing degrees of tilt (0 to 50 in 10° steps). We then observed the calculated roll values to closely track with actual values (See Figure S2).

*Figure S2. Demonstration showing angular data (specifically Roll) resolved from raw accelerometry. Bench test shows two devices attached to gimbal mount, while moving in Roll orientation 15° steps (from 0 to 120°), and repeated at increasing levels of tilt (0 to 50°, increments of 10°). Top subplot shows raw data from device #1. Middle subplot shows processed Roll data from device #1. Bottom subplot shows processed Roll data from device #2.*

Risk Indices: Development and Selection

Recognising the established relationship between lateral tilt and relevant maternal and fetal haemodynamics,^3-8^ we aimed to capture this through weighted risk indices that attribute exposure risk proportional to body roll. As such, in addition to the unweighted exposure measurements (i.e. time in broad supine and time in supine low-tilt – Risk Index #1 and Risk Index #2), several weighted risk indices were developed to investigate if weighted exposure risk provided stronger association with outcome variables of interest.

Two additional novel weighted risk exposure indices were selected for main analysis because they were deemed to closely align with current evidence on haemodynamic properties relative to body roll. Risk weighting #3 (Risk Index #3) builds upon studies that report minimal change in relevant haemodynamics with body roll between 0-|15|° (weight=1, |x| indicates both + and −), with haemodynamic improvement as tilt increases (decreasing risk weighting), and is considered similar to lateral when roll ≥|45|° (weight=0) (Figure 2D). Risk weighting #4 (Risk Index #4) is inspired by the haemodynamic contrast between supine (0°) and lateral (|90|°) positions and describes relative exposure across this range through a linear decaying relationship (weight=1 at 0°, weight=0 at 90°, Figure 2D).

Subsequent risk weightings were either simple variations of the two primary novel indices, or combined components of each to form unique exposure profiles, while still being founded upon the values presented in literature. As illustrated in Figure S3, Risk Index A, B and C are simple variations on Risk Index #4 in the main text, where risk weight = 1 at 0°, and risk decays linearly to zero at the denoted thresholds of 45°, 60° and 75° respectively, and remains zero thereafter (see Figure S3A-C). Risk Index D captures an unweighted exposure measurement where time spent between 0° and 30° has a risk weight = 1 and risk weight = 0 at >30°. Risk Index E, F and G capture risk as the highest value (i.e. weight=1) for a (variable) small range about 0° (15°, 30° and 15° respectively), before decaying to zero at variable thresholds (45°, 60° and 75° respectively; see Figure S3D-F).

*Figure S3. Diagrammatic representation of exposure risk indices used in additional analyses. A = Risk Index A, B = Risk Index B, C = Risk Index C, D = Risk Index D, E = Risk Index E, F = Risk Index F, G = Risk Index G.*

# Supplementary Results

## Night-to-Night Variability

Time spent in the supine position each night varied considerably within and between participants. For the broad definition of supine (0-45*°*; Figure S4A), half of nights (282/563 = 50.1%) contained at least one hour and just over a quarter of nights (146/563 = 25.9%) contained over two hours. Only 3 women did not sleep in broad supine at all during the week, whereas 42/85 (49.4%) women spent at least 10 minutes in broad supine every night. The median difference between the minimum and maximum amount of broad supine time across the week within person was 97.5 minutes (61.9, 143.0), with the median standard deviation of 35.6 minutes (24.0, 52.5).

For supine low-tilt positioning (0-15°; Figure S4B), only 75 (13.3%) nights contained more than one-hour (across 30 women) and 25 (4.4%) nights contained more than two hours (across 13 women). A third of women (28/85 = 32.9%) did not sleep at a low supine tilt on any night of the monitoring period, whereas only 7 women (8.2%) spent at least 10 minutes in supine low-tilt positioning every night. The median difference between the minimum and maximum amount of supine low-tilt time across the week within person was 37.5 minutes (3.8, 71.3), with the median standard deviation of 14.4 minutes (1.5, 27.5).

Simulation revealed that precision of supine measurement degraded as the number of nights of monitoring decreased, with an absolute difference of 35.4 (95% CI 26.4, 44.4) minutes between the first night measurement and the average of seven nights for broad supine (0-45*°*), and an absolute difference of 17.0 (95% CI 9.7, 24.3) minutes for supine low-tilt (0-15*°*; Fig S5).

*Figure S4. Hours per night of supine positioning defined as A) 0-45° and B) 0-15° with each vertical line and associated dots representing each night for each participant. Participants are plotted in same position on x axis on each figure, from lowest to highest average time supine 0-45° per night. Orange dots represent median for each participant.*

*Figure S5. Absolute difference in supine duration overnight between the mean of seven nights monitored within each participant versus the mean of a reduced number of nights, for Broad Supine and Supine Low-Tilt. The number of nights monitored is chronological for each participant i.e. 1 = the first night monitored, 4 = mean of the first four nights monitored. The absolute difference from mean supine duration over seven nights increases as the number of nights of monitoring decreases, indicating that fewer nights of monitoring provide less precision.*

## Impact of pillows on overnight body position

Eighty-two women reported on pillow use during the study. Only 5 women (6%) did not use extra pillows than those under their head for their week of monitoring, compared to 23 women (28%) who used additional pillows between the legs and/or supporting the abdomen every night, and 33 women (40%) who included pillows to support behind their back every night. The remaining 21 women (26%) varied their nightly pillow use throughout the monitoring period.

To determine whether overnight body position was affected by commercially available pregnancy pillows, specifically those which are positioned to support side sleeping, linear mixed modelling with type of pillow use as the fixed factor and participant as random factor were performed to compare duration of supine monitoring time overnight. For this analysis, duration of supine position for 0-45° was square root transformed and duration of supine position for 0-15° was log transformed due to extreme skewness.

Using pillows behind the back did not have an effect on the amount of time spent in the supine position (0-45°; 78.6 (21.8, 136.2) mins) compared to using other additional pillows (41.5 (6.4, 104.4) mins, p = .25) or no additional pillows (65.5 (13.0, 137.7 mins), p = .92; Figure S6A).

Using pillows behind the back did not significantly impact on low-tilt supine positioning (0-15°) either, with 0.6 (0.1, 23.7) minutes observed compared to 0.6 (0.2, 21.1) minutes when using other additional pillows (p = .90) and 6.4 (0.2, 30.7) minutes with no additional pillows (p = .14; Figure S6B).

B

A

*Figure S6 – Time spent in the supine position defined as A) 0-45 degrees and B) 0-15 degrees, for nights using a pillow to support the back (n = 256), nights using other additional pillows between the knees/under the abdomen (n = 217) and nights using no additional pillows (n = 63).*

## Data Quality Control - belt versus tape

As part of this study, we also investigated the feasibility and reliability of an alternate device application, i.e., taped to the abdomen rather than applied via the elasticised belt. Here we attempt to represent the discrepancy between the two devices, whilst accounting for their necessary yet subtly different positioning on the body at the time of recording. This was approached via the following stepwise procedure:

1) Extract data from ‘belt’ and ‘tape’ devices and create two time series of roll data.
2) Clip data to ‘monitoring time’, representing the overnight period where both devices were worn simultaneously.
3) To adjust for time drift and device time calibration being subtly different between devices, we align the two signals using the lag identified via cross-correlation of two discrete-time series (MATLAB function ‘xcorr’).
4) To assess the overall strength of the relationship between the two devices, we calculated **Spearman’s** **rho**, as data was not normally distributed. While this rank-based metric reports the association between two series, it does not capture offset.
5) To describe the offset between the two devices, we use simple **mean difference** between the two time series.
6) To assess the variability between the two devices, we calculated the **mean absolute error** between the two time series.

Overall, when comparing values obtained from belt applied versus taped devices, the group average (mean) for Spearman's rho (i.e., rank correlation coefficient) across all participants was 0.93, the average mean difference was 0.72°, and the average mean absolute error was 9.26°. Device preference was mixed, with 50% of participants reporting a preference for belt over tape. Table S1 provides per participant details on belt versus tape placement of the monitoring device.

Table S1 – Per participant details on belt versus tape placement of monitoring device.

| ID | Spearman’s rho | Mean Difference (°) | Mean Absolute Error (°) | Pt Device Preference |
| --- | --- | --- | --- | --- |
| 1 | - | - | - | Belt |
| 2 | 0.87 | -2.10 | 17.85 | Tape |
| 3 | 0.94 | -9.42 | 9.98 | Belt |
| 4 | 0.96 | 8.20 | 8.73 | Tape |
| 5 | 0.99 | 4.55 | 4.95 | Tape |
| 6 | 0.92 | 2.10 | 7.39 | Tape |
| 7 | 0.96 | -4.67 | 10.82 | Tape |
| 8 | 0.86 | -4.03 | 7.01 | Belt |
| 9 | 0.90 | 5.81 | 8.73 | Belt |
| 10 | 0.86 | 6.34 | 7.00 | Belt |
| 11 | 0.94 | -5.10 | 12.24 | Tape |
| 12 | 0.96 | 6.19 | 7.13 | Belt |

*Note*. ID 1 taped device was worn but did not record. Pt = participant.

Table S2. Relationship between Supine Risk Index #1 (duration broad supine per night 0-45°) and fetal growth outcomes

|  | Customised Birthweight Centile | | | | Birthweight (g) | | | | Gestational Age at Delivery (days) | | | |
| --- | --- | --- | --- | --- | --- | --- | --- | --- | --- | --- | --- | --- |
|  | Model 1 | | Model 2 | | Model 1 | | Model 2 | | Model 1 | | Model 2 | |
|  | B (SE) | β | B (SE) | β | B (SE) | β | B (SE) | β | B (SE) | β | B (SE) | β |
| Supine (mins) | -1.5 (3.21) | -.05 | -1.50 (3.13) | -.05 | -11.36 (49.63) | -.03 | 2.81 (48.18) | .02 | 1.29 (1.06) | 0.13 | 1.25 (1.05) | .13 |
| Age (yrs) |  |  | -0.32 (0.74) | -.05 |  |  | -11.01 (11.21) | -.11 |  |  | -0.10 (0.24) | -.04 |
| Nulliparous |  |  | -18.26 (6.56) | -.31** |  |  | -304.54 (99.27) | -0.34** |  |  | 1.67 (2.16) | .09 |
| GDM |  |  | 6.46 (8.67) | .08 |  |  | -161.63 (131.44) | -.13 |  |  | -5.07 (2.86) | -.19^#^ |
| HTN |  |  | 20.63 (11.61) | .19^#^ |  |  | -80.59 (177.07) | -.05 |  |  | -10.03 (3.85) | -0.29* |
| BMI |  |  |  |  |  |  | 8.39 (7.34) | .12 |  |  | -0.09 (0.16) | -.06 |
| F |  | 0.22 |  | 1.90 |  | .05 |  | 2.41* |  | 1.48 |  | 2.20^#^ |
| R^2^ (%) |  | 0.27 |  | 5.14 |  | 0.06 |  | 9.22 |  | 1.77 |  | 7.97 |

*** p < .01, *p < .05, ^#^ p< .1. Note. Adjusted for age, parity, GDM, HTN and BMI (customised centile not adjusted for BMI). R^2^ adjusted for Model 2. Supine (mins) was Box Cox transformed and standardised to z scores.*

Table S3. Relationship between Supine Risk Index #2 (duration supine low-tilt per night 0-15°) and fetal growth outcomes

|  | Customised Birthweight Centile | | | | Birthweight (g) | | | | Gestational Age at Delivery (days) | | | |
| --- | --- | --- | --- | --- | --- | --- | --- | --- | --- | --- | --- | --- |
|  | Model 1 | | Model 2 | | Model 1 | | Model 2 | | Model 1 | | Model 2 | |
|  | B (SE) | β | B (SE) | β | B (SE) | β | B (SE) | β | B (SE) | β | B (SE) | β |
| Supine (mins) | 1.56 (3.21) | .05 | -.01 (3.2) | .00 | 36.63 (49.48) | .08 | 23.53 (48.3) | .05 | 1.15 (1.07) | .12 | 1.54 (1.05) | .16 |
| Age (yrs) |  |  | -0.28 (0.74) | -.04 |  |  | -10.41 (11.23) | -.10 |  |  | -0.08 (0.24) | -.04 |
| Nulliparous |  |  | -18.3 (6.63) | -.31** |  |  | -297.71 (100.06) | -.33** |  |  | 2.16 (2.17) | 0.11 |
| GDM |  |  | 6.31 (8.68) | .08 |  |  | -159.74 (131.14) | -.13 |  |  | -4.81 (2.84) | -.18^#^ |
| HTN |  |  | 20.42 (11.83) | .20^#^ |  |  | -96.67 (179.78) | -.06 |  |  | -10.83 (3.90) | .-.31** |
| BMI |  |  |  |  |  |  | 8.45 (7.22) | .12 |  |  | -0.11 (0.16) | -.08 |
| F |  | 0.24 |  | 1.85 |  | 0.55 |  | 2.45* |  | 1.17 |  | 2.34* |
| R^2^ (%) |  | 0.29 |  | 4.86 |  | 0.66 |  | 9.50 |  | 1.40 |  | 8.83 |

*** p < .01, *p < .05, ^#^ p< .1. Note. Adjusted for age, parity, GDM, HTN and BMI (customised centile not adjusted for BMI). R^2^ adjusted for Model 2. Supine (mins) was Box Cox transformed and standardised to z scores.*

Table S4. Relationship between Supine Risk Index #3 and fetal growth outcomes

|  | Customised Birthweight Centile | | | | Birthweight (g) | | | | Gestational Age at Delivery (days) | | | |
| --- | --- | --- | --- | --- | --- | --- | --- | --- | --- | --- | --- | --- |
|  | Model 1 | | Model 2 | | Model 1 | | Model 2 | | Model 1 | | Model 2 | |
|  | B (SE) | β | B (SE) | β | B (SE) | β | B (SE) | β | B (SE) | β | B (SE) | β |
| Risk Index #3 | -0.29 (3.21) | -.01 | -0.21 (3.15) | -.01 | 26.14 (49.56) | .06 | 30.18 (48.03) | .07 | 1.60 (1.06) | .17 | 1.64 (1.04) | .17 |
| Age (yrs) |  |  | -0.29 (0.74) | -.04 |  |  | -10.30 (11.20) | -.10 |  |  | -0.09 (0.24) | -.04 |
| Nulliparous |  |  | -18.35 (6.57) | -.31** |  |  | -302.06 (99.07) | -.33** |  |  | 1.85 (2.15) | .09 |
| GDM |  |  | 6.30 (8.68) | .08 |  |  | -160.27 (130.97) | -.13 |  |  | -4.86 (2.84) | -.18^#^ |
| HTN |  |  | 20.49 (11.67) | .20^#^ |  |  | -92.78 (177.46) | -.06 |  |  | -10.44 (3.84) | -0.30** |
| BMI |  |  |  |  |  |  | 9.01 (7.29) | .13 |  |  | -0.09 (0.16) | -.06 |
| F |  | 0.01 |  | 1.85 |  | .28 |  | 2.48* |  | 2.30 |  | 2.40* |
| R^2^ (%) |  | 0.00 |  | 0.05 |  | 0.00 |  | 0.10 |  | 0.03 |  | 0.09 |

*** p < .01, *p < .05, ^#^ p< .1. Note. Adjusted for age, parity, GDM, HTN and BMI (customised centile not adjusted for BMI). R^2^ adjusted for Model 2. Risk Index #3 was Box Cox transformed and standardised to z scores.*

Table S5. Relationship between Supine Risk Index #4 and fetal growth outcomes

|  | Customised Birthweight Centile | | | | Birthweight (g) | | | | Gestational Age at Delivery (days) | | | |
| --- | --- | --- | --- | --- | --- | --- | --- | --- | --- | --- | --- | --- |
|  | Model 1 | | Model 2 | | Model 1 | | Model 2 | | Model 1 | | Model 2 | |
|  | B (SE) | β | B (SE) | β | B (SE) | β | B (SE) | β | B (SE) | β | B (SE) | β |
| Risk Index #4 | -2.48 (3.20) | -.09 | -2.65 (3.17) | -.09 | -48.16 (49.36) | -.11 | -19.26 (48.56) | -.04 | 0.18 (1.07) | 0.02 | 0.54 (1.06) | .06 |
| Age (yrs) |  |  | -0.33 (0.73) | -.05 |  |  | -11.36 (11.17) | -.11 |  |  | -0.12 (0.25) | -.05 |
| Nulliparous |  |  | -17.94 (6.56) | -.31** |  |  | -301.65 (99.40) | -.33** |  |  | 1.64 (2.18) | .08 |
| GDM |  |  | 6.94 (8.67) | .09 |  |  | -156.29 (131.76) | -.13 |  |  | -5.05 (2.89) | -.19^#^ |
| HTN |  |  | 21.82 (11.69) | .21^#^ |  |  | -68.48 (178.90) | -.04 |  |  | -10.06 (3.92) | -0.29* |
| BMI |  |  |  |  |  |  | 7.91 (7.29) | .12 |  |  | -0.11 (0.16) | -.08 |
| F |  | 0.60 |  | 2.01^#^ |  | 0.95 |  | 2.44* |  | 0.03 |  | 1.98^#^ |
| R^2^ (%) |  | 0.73 |  | 5.71 |  | 1.15 |  | 9.41 |  | 0.03 |  | 6.59 |

*** p < .01, *p < .05, ^#^ p< .1. Note. Adjusted for age, parity, GDM, HTN and BMI (customised centile not adjusted for BMI). R^2^ adjusted for Model 2. Risk Index #4 was Box Cox transformed and standardised to z scores.*

Table S6. Regression coefficients for predicting customised birthweight centile from supplementary risk indices of supine position

|  | Unadjusted | |  | Adjusted | |  |
| --- | --- | --- | --- | --- | --- | --- |
|  | Coeff (95% CI) | Beta | p value | Coeff (95% CI) | Beta | p value |
| Risk Index A | 0.56 (-5.83, 6.94) | .02 | .86 | -0.02 (-6.30, 6.26) | -.00 | 1.00 |
| Risk Index B | -1.70 (-8.08, 4.67) | -.06 | .60 | -1.79 (-8.02, 4.44) | -.06 | .57 |
| Risk Index C | -3.11 (-9.46, 3.25) | -.11 | .33 | -2.87 (-9.10, 3.35) | -.10 | .36 |
| Risk Index D | 1.67 (-4.71, 8.05) | .06 | .60 | 0.94 (-5.33, 7.23) | .03 | .77 |
| Risk Index E | -0.84 (-7.22, 5.55) | -.03 | .80 | -0.99 (-7.23, 5.25) | -.03 | .75 |
| Risk Index F | -2.46 (-8.82, 3.90) | -.08 | .44 | -2.27 (-8.49, 3.95) | -.08 | .47 |
| Risk Index G | -3.09 (-9.44, 3.26) | -.11 | .34 | -2.81 (-9.03, 3.41) | -.10 | .37 |

Note. Adjusted for age, nulliparity, gestational diabetes mellitus, hypertensive disorders of pregnancy. Risk Index variables Box Cox transformed and standardized to z-scores - coefficient represents change in customised birthweight centile given one SD increase in supine variable. See Fig S3 for definition of each Risk Index.

Table S7. Regression coefficients for predicting birthweight from supplementary risk indices of supine position

|  | Unadjusted | |  | Adjusted | |  |
| --- | --- | --- | --- | --- | --- | --- |
|  | Coeff (95% CI) | Beta | p value | Coeff (95% CI) | Beta | p value |
| Risk Index A | 29.49 (-69.06, 128.04) | .07 | .55 | 32.15 (-63.54, 127.83) | .07 | .51 |
| Risk Index B | -0.89 (-99.65, 97.87) | -.00 | .99 | 12.01 (-83.38, 107.41) | .03 | .80 |
| Risk Index C | -32.49 (-131.00, 66.01) | -.07 | .51 | -11.22 (-106.00, 83.54) | -.02 | .81 |
| Risk Index D | 46.87 (-51.35, 145.10) | .10 | .35 | 47.43 (-47.58, 142.44) | .11 | .32 |
| Risk Index E | 2.69 (-96.07, 101.45) | .01 | .96 | 14.09 (-81.69, 109.88) | .03 | .77 |
| Risk Index F | -15.07 (-113.78, 83.63) | -.03 | .76 | 1.59 (-93.65, 96.83) | .00 | .97 |
| Risk Index G | -33.94 (-132.42, 64.54) | -.08 | .50 | -12.32 (-107.04, 82.41) | -.03 | .80 |

Note. Adjusted for age, nulliparity, gestational diabetes mellitus, hypertensive disorders of pregnancy and body mass index. Risk Index variables Box Cox transformed and standardized to z-scores - coefficient represents change in birthweight (g) given one SD increase in supine variable. See Fig S3 for definition of each Risk Index.

Table S8. Regression coefficients for predicting gestational age at delivery from supplementary risk indices of supine position

|  | Unadjusted | |  | Adjusted | |  |
| --- | --- | --- | --- | --- | --- | --- |
|  | Coeff (95% CI) | Beta | p value | Coeff (95% CI) | Beta | p value |
| Risk Index A | 1.58 (-0.52, 3.68) | .16 | .14 | 1.63 (-0.45, 3.70) | .17 | .12 |
| Risk Index B | 1.56 (-0.54, 3.67) | .16 | .14 | 1.58 (-0.48, 3.64) | .16 | .13 |
| Risk Index C | 0.88 (-1.25, 3.00) | .09 | .41 | 1.04 (-1.03, 3.11) | .11 | .32 |
| Risk Index D | 1.60 (-0.51, 3.70) | .16 | .14 | 1.74 (-0.32, 3.80) | .18 | .10 |
| Risk Index E | 1.45 (-0.66, 3.56) | .15 | .18 | 1.43 (-0.65, 3.51) | .15 | .17 |
| Risk Index F | 1.43 (-0.68, 3.54) | .15 | .18 | 1.42 (-0.65, 3.48) | .15 | .18 |
| Risk Index G | 0.85 (-1.28, 2.97) | .09 | .43 | 1.00 (-1.07, 3.07) | .10 | .34 |

Note. Adjusted for age, nulliparity, gestational diabetes mellitus, hypertensive disorders of pregnancy and body mass index. Risk Index variables Box Cox transformed and standardized to z-scores - coefficient represents change in gestational age at delivery (days) given one SD increase in supine variable. See Fig S3 for definition of each Risk Index.

Table S9. Regression coefficients for predicting 21^st^ INTERGROWTH birthweight centile from key indices of supine position overnight

|  | Unadjusted | |  | Adjusted | |  |
| --- | --- | --- | --- | --- | --- | --- |
|  | Coeff (95% CI) | Beta | p value | Coeff (95% CI) | Beta | p value |
| 0-45° supine min/night (Risk Index #1) | -2.17 (-8.00, 3.66) | -.08 | .46 | -0.94 (-6.50, 4.61) | -.04 | .74 |
| 0-15° supine min/night (Risk Index #2) | 1.26 (-4.58, 7.10) | .05 | .67 | -0.08 (-5.67, 5.50) | -.00 | .98 |
| Risk index #3 | 0.05 (-5.79, 5.90) | .00 | .99 | 0.48 (-5.08, 6.03) | -.02 | .86 |
| Risk Index #4 | -2.64 (-8.46, 3.18) | -.10 | .37 | -1.27 (-6.78, 4.24) | -.05 | .65 |

Note. Adjusted for age, nulliparity, gestational diabetes mellitus, hypertensive disorders of pregnancy and body mass index. Risk Index variables Box Cox transformed and standardized to z-scores - coefficient represents change in centile given one SD increase in supine variable. See Fig 1D for definition of each Risk Index. The Mdn (IQR) for 21^st^ INTERGROWTH birthweight centile was 71.5 (45.2, 87.8)%.

**B**

**A**

**C**

*Fig S7. Change in customised birthweight centile per week between growth scan and birth, with no relationship with average time spent in A) Risk Index #1 Broad Supine = 0-45° or B) Risk Index #2 Supine Low-Tilt = 0-15° overnight; and C) no difference in growth trajectory between the participants with above-median (≥ 66 min = High Supine) versus below-median (<66 min = Low Supine) duration in the supine (0-45°) position (change in customised birthweight centile per week = -1.4 (3.0) vs -3.3 (3.2), p = .07). Supine mins were Box Cox transformed and standardised to z scores.*

**References**

1.	Tan C-W, Park S. Design of accelerometer-based inertial navigation systems. IEEE Trans Instrum Meas. 2005; 54 (6): 2520-2530.

2.	Pedley M. Tilt Sensing Using a Three-Axis Accelerometer. Austin, TX: Freescale Semiconductor, Inc.; 2013.

3.	Sonnino C, Frassanito L, Piersanti A, et al. Impact of maternal lateral tilt on cardiac output during caesarean section under spinal anaesthesia: a prospective observational study. BMC Anesthesiol. 2022; 22 (1): 103.

4.	Armstrong S, Fernando R, Columb M, Jones T. Cardiac index in term pregnant women in the sitting, lateral, and supine positions: an observational, crossover study. Anesth Analg. 2011; 113 (2): 318-322.

5.	Ellington C, Katz VL, Watson WJ, Spielman FJ. The effect of lateral tilt on maternal and fetal hemodynamic variables. Obstet Gynecol. 1991; 77 (2): 201-203.

6.	Fujita N, Higuchi H, Sakuma S, Takagi S, Latif M, Ozaki M. Effect of right-lateral versus left-lateral tilt position on compression of the inferior vena cava in pregnant women determined by magnetic resonance imaging. Anesth Analg. 2019; 128 (6): 1217-1222.

7.	Couper S, Clark A, Thompson JMD, et al. The effects of maternal position, in late gestation pregnancy, on placental blood flow and oxygenation: an MRI study. J Physiol. 2021; 599 (6): 1901-1915.

8.	Humphries A, Mirjalili SA, Tarr GP, Thompson JMD, Stone P. The effect of supine positioning on maternal hemodynamics during late pregnancy. J Matern Fetal Neonatal Med. 2019; 32 (23): 3923-3930.
